# P2X7 receptor activation awakes a dormant stem cell niche in the adult spinal cord

**DOI:** 10.3389/fncel.2023.1288676

**Published:** 2023-12-18

**Authors:** María Victoria Falco, Gabriela Fabbiani, Cecilia Maciel, Spring Valdivia, Nathalia Vitureira, Raúl E. Russo

**Affiliations:** ^1^Departamento de Neurofisiología Celular y Molecular, Instituto de Investigaciones Biológicas Clemente Estable, Montevideo, Uruguay; ^2^Departamento de Fisiología, Facultad de Medicina, Universidad de la República, Montevideo, Uruguay

**Keywords:** ependymal cells, endogenous progenitors, P2X7 receptors, spinal cord injury, regeneration, purinergic signaling, BzATP, P2X7 receptor knockout mice

## Abstract

The ependyma of the spinal cord is a latent stem cell niche that is reactivated by injury, generating new cells that migrate to the lesion site to limit the damage. The mechanisms by which ependymal cells are reactivated after injury remain poorly understood. ATP has been proposed to act as a diffusible “danger signal” to alert about damage and start repair. Indeed, spinal cord injury (SCI) generates an increase in extracellular ATP around the lesion epicenter that lasts for several hours and affects the functional outcome after the damage. The P2X7 receptor (P2X7r) has functional properties (e.g., low sensitivity for ATP, high permeability for Ca^2+^) that makes it a suitable candidate to act as a detector of tissue damage. Because ependymal cells express functional P2X7r that generate an inward current and regenerative Ca^2+^ waves, we hypothesize that the P2X7r has a main role in the mechanisms by which progenitor-like cells in the ependyma react to tissue damage. To test this possibility, we simulated the P2X7r activation that occurs after SCI by *in vivo* intraspinal injection of the selective agonist BzATP nearby the central canal. We found that BzATP rescued ependymal cells from quiescence by triggering a proliferative response similar to that generated by injury. In addition, P2X7r activation by BzATP induced a shift of ependymal cells to a glial fibrillary acidic protein (GFAP) phenotype similar to that induced by injury. However, P2X7r activation did not trigger the migration of ependyma-derived cells as occurs after tissue damage. Injection of BzATP induced the expression of connexin 26 (Cx26) in ependymal cells, an event needed for the proliferative reaction after injury. BzATP did not induce these changes in ependymal cells of P2X7^–/–^ mice supporting a specific action on P2X7r. *In vivo* blockade of P2X7r with the potent antagonist AZ10606120 reduced significantly the injury-induced proliferation of ependymal cells. Our data indicate that P2X7r has a key role in the “awakening” of the ependymal stem cell niche after injury and suggest purinergic signaling is an interesting target to improve the contribution of endogenous progenitors to repair.

## Introduction

Signaling via neurotransmitters play a key role during development of neural circuits (Ben-Ari and Spitzer, 2010). ATP acting on ionotropic P2X (1–7) (Surprenant and North, 2009) or metabotropic P2Y (1, 2, 4, 6, 11-14) receptors (Abbracchio et al., 2006) regulates processes such as progenitor cell proliferation, migration, differentiation and synapse formation (Zimmermann, 2011). For example, during cortical development ATP released through connexin (Cx) hemichannels in radial glia (RG) activates P2Y1 receptors triggering Ca^2+^ waves that propagate among neighboring cells to control the proliferation of RG (Weissman et al., 2004). The Ca^2+^ signaling via purinergic receptors is also involved in the migration of intermediate neuronal progenitors to the subventricular zone of the adult brain (Liu et al., 2008).

Besides its myriad functions in health, ATP signaling plays a pivotal role in different pathologies with either beneficial or detrimental actions (Khakh and Alan North, 2006). Although both P2X and P2Y receptors seem to be involved in these events, the P2X7 receptor (P2X7r) merits particular attention. For instance, the P2X7r has a low sensitivity (activated at 100 μM to 3 mM of ATP), is highly permeable to Ca^2+^ and when activated for several seconds becomes permeable to large cations (Surprenant et al., 1996). Thus, the unique functional properties of the P2X7r make it a suitable candidate to act as a detector of tissue damage generating profound effects in a variety of cell types in the central nervous system (Lecca et al., 2016).

Increased levels of extracellular ATP are related to different kinds of tissue insults (Cauwels et al., 2014). For example, ATP levels after spinal cord injury (SCI) increase around the lesion epicenter for several hours (Wang et al., 2004). ATP may act as a diffusible “danger signal” to alert about damage and start repair (Abbracchio et al., 2009), although purinergic signaling has been also implied in the expansion of tissue damage after SCI (Wang et al., 2004; Davalos et al., 2005; Peng et al., 2009). Blockade of P2X7r has been reported to improve functional recovery after SCI by reducing the size of the lesion, microglial activation and reactive astrocytosis (Wang et al., 2004; Davalos et al., 2005), suggesting P2X7r and downstream cellular events (e.g., Ca^2+^ waves) as relevant targets to treat SCI.

The ependyma of the spinal cord (SC) -a latent stem cell niche that is reactivated by injury (Sabelström et al., 2013; Moreno-Manzano, 2020)- bears different kinds of purinergic receptors. Cerebrospinal fluid contacting neurons have functional P2X2 receptors that generate a powerful excitation upon activation by ATP (Marichal et al., 2009). Cultured ependymal progenitors express P2X4/P2X7 and P2Y1/P2Y4 receptors with downregulation of P2Y1 and upregulation of P2Y4 in ependymal progenitors obtained from the injured cord (Gómez-Villafuertes et al., 2015). In SC slices, we previously showed that ependymal cells express P2X7r and that the selective agonist BzATP applied in different domains of the central canal (CC) generates an inward current and propagated Ca^2+^ waves (Marichal et al., 2016).

We hypothesize here that the P2X7r has a main role in the mechanisms by which progenitor-like cells in the ependyma react to tissue damage. We simulated the P2X7r activation that would occur after SCI by *in vivo* intraspinal injection of BzATP nearby the CC. We found that BzATP rescued ependymal cells from quiescence by triggering a proliferative response similar to that generated by injury. In addition, similar to injury (Trujillo-Cenóz et al., 2021), BzATP induced a shift of ependymal cells to a glial fibrillary acidic protein (GFAP) phenotype and the expression of Cx26. However, P2X7r activation did not trigger the migration of ependyma-derived cells observed after injury (Meletis et al., 2008). Together, our data indicate that purinergic signaling has a key role in the molecular mechanisms by which injury reactivates the ependymal stem cell niche.

## Materials and methods

### Animals

Adult (P90-150) C57BL/6J (The Jackson Laboratories, RRID:IMSR_JAX:000664), FoxJ1CreER-R26RtdTomato (kind gift of Prof. Jonas Frisén, Karolinska Institutet, see Llorens-Bobadilla et al., 2020), GFAP-GFP (kind gift of Prof. Frank Kirchoff, Saarland University, see Nolte et al., 2001) and P2X7r knock out (B6.129P2-P2rx7tm1Gab/J, The Jackson Laboratories; RRID:IMSR_JAX:005576) mice were used. Some experiments were done in mice obtained from the crossing of these transgenic lines. To induce the expression of tdTomato (tdT) in adult mice we injected Tamoxifen (Sigma; 2 mg, 20 mg/ml in corn oil, i.p.) for 5 days and allowed 5 days between the last injection and surgery to ensure clearance (Meletis et al., 2008). All experimental procedures were approved by our local Committee for Animal Care (protocol #005/09/2015).

### Spinal cord injury

Animals were sedated with diazepam (5 mg/kg, i.p.) and anesthetized with ketamine (100 mg/kg, i.p) and xylazine (10 mg/kg, i.p.). Injury of the dorsal aspect of the SC was performed as described by Frisén et al. (1993). Briefly, after laminectomy the dorsal funiculus at low thoracic level (T13) was cut transversely with microsurgical scissors (depth ∼0.8 mm) and the lesion was extended rostrally to comprise about one SC segment. Recovery from anesthesia was promoted with flumazenil (0.5 mg/kg, i.p.) and yohimbine (2 mg/kg, i.p.). Tramadol (3 mg/kg, i.p) was administered for pain relief. A second dose of tramadol was applied 24 h after surgery. Sham injured animals were used as controls by performing all the procedures described above but without injuring the cord.

### *In vivo* activation of P2X7r

Intraspinal injection of the selective P2X7r agonist BzATP (1 μl, 1 mM in saline) was performed with a glass micropipette (∼40 μm tip) pulled from graduated glass capillaries (Hirschmann ring caps 9600105). The glass micropipette was secured in a holder attached to a digital micromanipulator and lowered 550 μm lateral from the midline to a depth of 700 μm in 20 μm steps to minimize tissue distortion. The drug was injected at a speed of 0.1 μl/min. After the injection, the pipette was withdrawn in 20 μm steps at 15 s intervals. To visualize the extent of tissue affected by the injection we added fluorescein (0.02 g/ml) to the solution. Injections of vehicle without BzATP served as control.

### Immunohistochemistry

Animals were anesthetized with ketamine (100 mg/kg, i.p), xylazine (10 mg/kg, i.p.) and diazepam (10 mg/kg, i.p.) and fixed by intracardiac perfusion with 4% paraformaldehyde in 0.1 M phosphate buffer (PB). The following primary antibodies were used: anti-BrdU (mouse monoclonal, 1:20, DSHB, #G3G4), anti-GFAP (1:20, DSHB. #8-1E7), anti-Ki67 (rabbit polyclonal, 1:500; Abcam, #AB15580), anti-Cx26 (rabbit polyclonal, 1:200; Alomone Labs, #ACC-212) and anti-P2X7r (rabbit polyclonal, 1:100, Alomone Labs, #APR004). The SC was sectioned with a vibrating microtome (60–80 μm thick), washed twice in PBS and then incubated with primary antibodies in PBS with 0.3% Triton X-100 (Sigma-Aldrich). Sections were then incubated in secondary antibodies conjugated with different fluorophores. Nuclei were stained with DAPI (Invitrogen). Negative control experiments were performed omitting primary antibodies.

### Proliferation assay

Injury-induced proliferation peaks between day 3 and 7 after injury to subside to day 14, 9 when it stills remains higher than control (Mothe and Tator, 2005). Thus, to detect strongest proliferative response, BrdU (100 mg/kg, i.p.; Sigma) or EdU (40 mg/kg, i.p.; Biosynth Ltd.) were injected twice daily (4 h interval) from day 3 to day 5after BzATP injection or SCI. The animals were anesthetized as already described at day 8 after injury or BzATP injection and perfused with 4% paraformaldehyde in 0.1 M phosphate buffer (PB, pH 7.4). To visualize BrdU labeling, sections were pretreated with 2 N HCl, washed several times with PB and incubated overnight with anti-BrdU. EdU labeling was revealed by using the Click-iT Alexa Fluor 647 imaging kit (Invitrogen, Thermo Fisher Scientific).

### *In vivo* blockade of P2X7r in the injured spinal cord

To block P2X7r that may be activated by fast mechanically induced ATP release (Xiong et al., 2018) and acute cell death upon traumatic injury, we made a preemptive blockade by an intraspinal injection of the specific, water soluble antagonist AZ10606120 (1 μl of a 1 μM solution) using the same coordinates as for injection of BzATP. Immediately after completion of the injection, we performed a transverse dorsal hemisection followed by injection within the lesion site of 50 μl of Pluronic F127 (20% w/v in saline at 5–10°C) containing AZ10606120 at a 10 μM concentration to block the sustained component of ATP release (Wang et al., 2004). Pluronic F127 dissolved in saline was injected within the injured SC as control.

### Experimental design and statistical analyses

Mice of either sex were randomly assigned to experimental groups. Animals injected with vehicle were used as controls. When using injured mice, we checked that loss of weight at sacrifice did not exceed 15%. Tissue within the fluorescein halo (∼1 mm) was selected for the analysis (70 μm sections as samples). All images obtained for quantitative analysis were taken using the same preset parameters and analyzed with ImageJ (ImageJ, National Institutes of Health). For quantitative analysis of immunofluorescence data, a minimum of 5 sections were analyzed and averaged per biological replicate (3–5 animals for each experimental condition, unless otherwise stated). To analyze changes in the expression of Cx26, the immunoreactive puncta were converted into particles using a macro developed in IJ1macro language and quantified in the area occupied by ependymal cells (0–20 μm from the CC lumen) within the injection site (± 250 μm, in 70 μm transverse sections). The confocal images were acquired with a LSM800 Zeiss Airyscan confocal microscope. Statistical analyses were performed with Graphpad prism 9.50.0 with statistical significance set at *p* < 0.05. The statistical test chosen for each experiment is noted next to the corresponding result. Numerical values are reported as mean ± SD.

## Results

**FIGURE 1:**
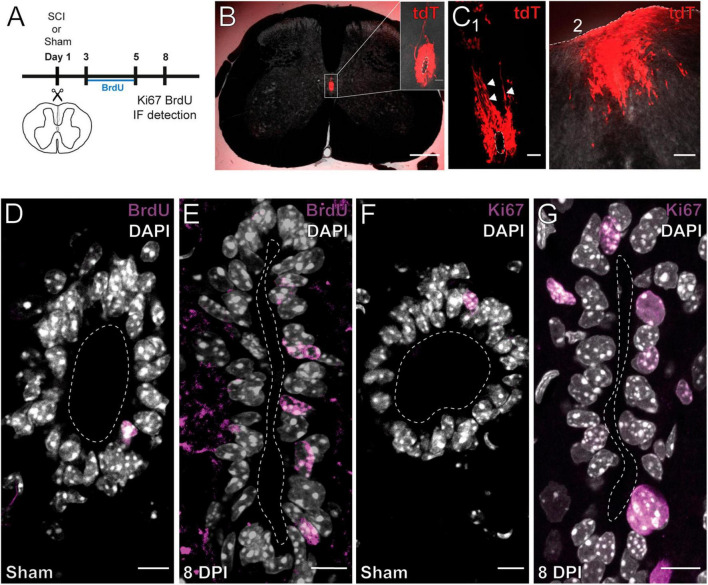
Injury induces the proliferation and migration of ependymal cells. **(A)** Experimental design. **(B)** Ependymal cells in the intact SC of a FoxJ1CreER-tdTomato mouse. The CC is shown at higher magnification (inset). **(C)** Ependymal cells of a 15 DPI FoxJ1CreER-tdTomato mouse appear to detach from the ependyma [**(C1)**, arrowheads] and reach the lesion site **(C2)**. **(D–H)** Immunoreactivity for BrdU and Ki67. The number of BrdU + **(D)** and Ki67 + **(G)** nuclei is higher in 8 DPI mice compared to sham injured animals [**(E,H)**, respectively]. Scale bars: **(B)**, main panel, 250 μm; inset 20 μm; **(C1)**, 50 μm, **(C2)**, 100 μm; **(D–G)**, 10 μm.

**FIGURE 2:**
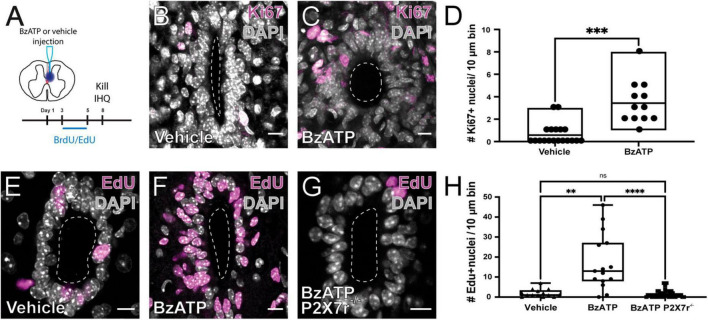
Activation of P2X7r by BzATP induces the proliferation of ependymal cells. **(A)** Experimental design. Immunoreactivity for Ki67 in the ependymal of mice injected with vehicle **(B)** and BzATP **(C)**. **(D)** Boxplot showing that there is a significant difference in the number of Ki67 + nuclei between vehicle and BzATP injected animals (unpaired *t*-test with Welch’s post-test correction, *p* = 0.0003). EdU + nuclei after injection of vehicle **(E)** and BzATP **(F)** in wild type mice. **(G)** The injection of BzATP in P2X7r^–/–^ mice does not promote the proliferation of ependymal cells. **(H)** Boxplot showing that the number of EdU + nuclei 8 days after vehicle (left) and BzATP (middle) was significantly different in wild type mice (*p* = 0.0014, Kruskal-Wallis test). BzATP injected in P2X7^–/–^ mice generated significantly fewer EdU + nuclei than in wild type mice (middle and right, Kruskal-Wallis test, *p* < 0.0001). Notice that the number of EdU + nuclei in P2X7^–/–^ mice was not statistically different than that of wild type animals injected with vehicle (left and right, *p* > 0.999). The line of the boxplots represents the median of the values for each sample. **p < 0.005; ***p < 0.001; and ****p < 0.0001. Scale bars: **(B,C,E–G)**, 10 μm.

**FIGURE 3:**
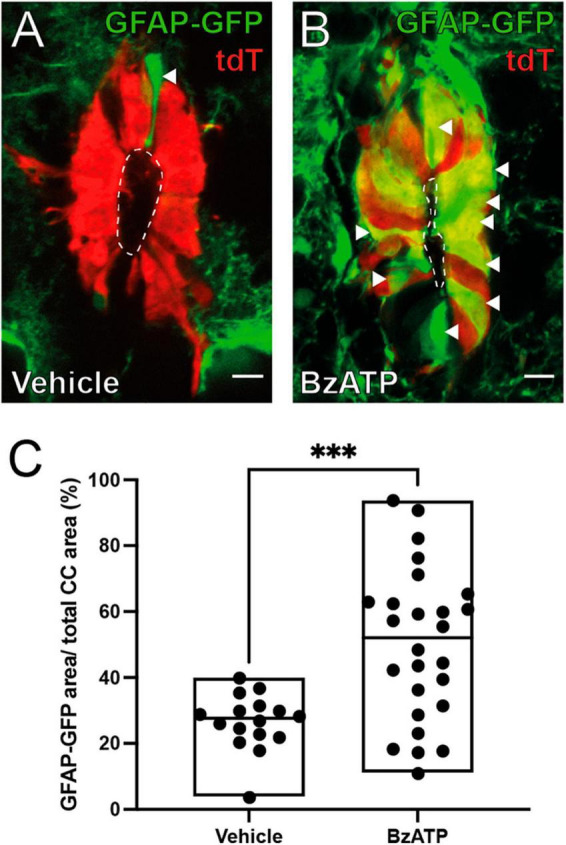
BzATP injection increases the expression of GFAP within the ependyma. **(A)** Ependymal cells (red) in FoxJ1CreER-tdTomato/GFAP-GFP mice 8 days after injection of vehicle. A few ependymal cells in the dorsal pole express GFP under the control of GFAP promoter (green, arrowhead). **(B)** After BzATP injection many cells in the ependymal cell layer express GFP indicating a GFAP phenotype (arrowheads). **(C)** Boxplot showing a statistically significant increase in the ratio of the area of GFAP-GFP over the total area of the ependyma between BzATP and vehicle injected animals (Mann-Whitney test, *p* = 0.0008). The line of the boxplots represents the median of the values for each sample. ***p < 0 001. Scale bars: **(A,B)** 10 μm.

As shown by others (Mothe and Tator, 2005; Meletis et al., 2008), SCI reactivates the ependymal stem cell niche. Figure 1A shows the experimental design to explore the reactivation of the stem cell niche after injury. In FoxJ1CreER-tdT mice, recombination induced by tamoxifen produces the expression of the reporter gene specifically in ependymal cells (Figure 1B and inset; see Meletis et al., 2008). At 15 days post-injury (DPI), tdT + cells close to the lesion epicenter seem to detach from the ependyma (Figure 1C1) to reach the lesion site (Figure 1C2). As described by Mothe and Tator (2005), injury induced the reactivation of ependymal cell proliferation as evidenced by the uptake of the thymidine analog BrdU (Figures 1D, E) and immunohistochemistry for Ki67 (Figures 1F, G). Because ATP around the lesion epicenter increases dramatically after injury (Wang et al., 2004) and ependymal cells have functional P2X7r (Marichal et al., 2016), we speculated that activation of P2X7r may be involved in triggering the initial reaction of ependymal cells to injury. To test this idea, we made intraspinal injections of the selective P2X7r agonist BzATP (1 mM, 1 μl) close to the CC (700 μm depth, 550 μm from midline) of adult mice (Figure 2A). Compared to the intraspinal injection of vehicle (Figure 2B), the number of Ki67 nuclei increased significantly 8 days after a single application of BzATP (Figures 2C, D, *p* = 0.0003, unpaired *t*-test with Welch’s post-test correction). Similarly, the number of EdU + nuclei after injection of BzATP was significantly higher than in control conditions (Figures 2E, F, H, *p* = 0.0014, Kruskal-Wallis test). Although BzATP is far more potent than ATP at P2X7r, it has also been shown to be an agonist of P2Y receptors (Boyer and Harden, 1989), a partial agonist of P2X4 (Bowler et al., 2003) and other purinergic receptors (Bianchi et al., 1999). To test whether the effect of intraspinal injection of BzATP can indeed be attributed to the activation of P2X7r, we used a P2X7r knockout mouse (Solle et al., 2001). Supplementary Figure 1 shows the validation of the P2X7 knockout mouse, with the lack of the P2X7r gene (A) and a negative immunoreactivity for the P2X7r (B) as compared with wild type mice (C). As shown in Figures 2G, H, application of BzATP close to the CC did not induce the proliferation of ependymal cells in P2X7r^–/–^ mice, supporting the idea that the effects induced by BzATP were indeed generated by the activation of P2X7r. Despite the resumption of proliferation induced by the injection of BzATP, we found no migration of tdT + ependyma-derived cells at 8 (see Figure 3B), 15 or 30 days (data not shown) after P2X7r activation.

The expression of the GFAP normally occurs in a subset of ependymal cells in the dorsal pole of the CC (Sabourin et al., 2009; Figure 3A, arrowhead) but appears in other quadrants of the ependyma after SCI (Trujillo-Cenóz et al., 2021). We found that injection of saline did not change the expression profile of GFAP (Figure 3A), but activation of P2X7r by BzATP induced a GFAP phenotypic change similar to that induced by injury (Figures 3B, C; *p* = 0.0008, Mann-Whitney test). Similar to the proliferative response, BzATP in P2X7r^–/–^ also failed to produce a change to a GFAP + phenotype (Supplementary Figure 2).

**FIGURE 4:**
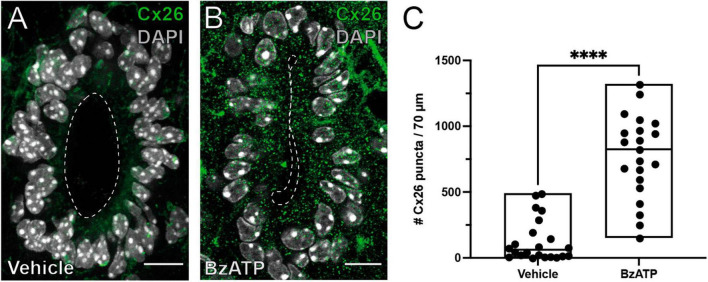
P2X7r activation induces the expression of Cx26. Immunoreactivity for Cx26 (green) in the ependyma of mice injected with vehicle **(A)** or BzATP **(B)**. **(C)** Plot showing the average number of Cx26 puncta in the ependyma within the injected spinal cord. Notice the number of Cx26 puncta is significantly higher in BzATP injected mice (Mann-Whitney test, *p* < 0.0001). The line of the boxplots represents the median of the values for each sample. ****p < 0.0001. Scale bars: **(A,B)**, 10 μm.

Connexin signaling is involved in the reactivation of the ependymal stem cell niche, with upregulation of Cx26 around the lesion epicenter (Fabbiani et al., 2020). We speculated that P2X7r activation may induce the re-expression of Cx26 associated with the resumption of proliferation in ependymal cells. To test this idea, we injected BzATP or saline and explored the expression of Cx26 in ependymal cells 8 days after P2X7r activation. After injection of saline, the expression of Cx26 in ependymal cells was very low as described previously in the intact adult ependyma (Fabbiani et al., 2020; Figure 4A). However, BzATP induced the expression of Cx26 in a way similar to that observed after dorsal hemisection of the SC (Figures 4B, C; *p* < 0.0001, Mann-Whitney).

**FIGURE 5:**
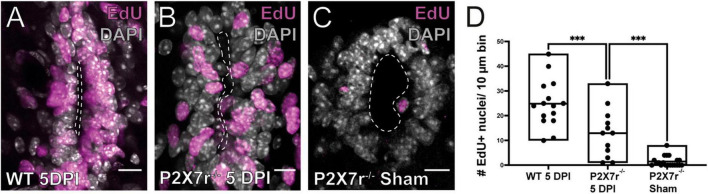
Injury-induced proliferation is reduced in P2X7r^–/–^ mice. The number of EdU+ nuclei within the ependyma of wild type (WT) mice **(A)** is higher than that of P2X^–/–^ **(B)** at 5DPI. **(C)** EdU + nuclei in the ependyma of sham injured P2X7r^–/–^ mice. **(D)** The boxplot shows a significant difference in the number of EdU + nuclei between P2X7r^–/–^ and WT mice at 5DPI (left and middle, ANOVA, *p* = 0.0005). Notice that there was still a significant difference between the number of EdU + nuclei between injured and sham control P2X7r^–/–^ mice (middle and right, ANOVA, *p* = 0.0004). The line of the boxplots represents the median of the values for each sample. ***p < 0.001. Scale bars: **(A–C)**, 10 μm.

Our results show that P2X7r activation *in vivo* can induce some of the phenomenology observed during the reactivation of the ependymal stem cell niche after SCI, suggesting a role of P2X7r in this process. To test this idea we compared the proliferative response induced by a dorsal hemisection of the SC in wild type and P2X7r^–/–^ mice. The number of EdU + nuclei after SCI was significantly smaller in P2X7r^–/–^ mice compared to wild type animals (Figures 5A, B, D, *p* = 0.0005, ANOVA). However, the number of EdU + cells in sham-injured P2X7^–/–^ mice was still significantly lower than in injured P2X7r^–/–^ mice (Figures 5C, D; *p* = 0.0004, ANOVA), suggesting that although P2X7r play a role in the reactivation of the ependymal stem cell niche there are other signaling pathways that also regulate the proliferation of ependymal cells after injury.

In line with the lack of modulation of proliferation by BzATP in P2X7^–/–^ mice, the expression of GFAP in the ependyma of P2X7r^–/–^ mice did not change after injury (Supplementary Figure 3). Immunohistochemistry for GFAP showed similar levels of expression in both injured and sham injured mice (Supplementary Figures 3A–C; *p* = 0.3026, Mann-Whitney test). Finally, Cx26 expression after injury was significantly lower in P2X7r^–/–^ mice compared to WT mice (Supplementary Figures 4A–C; *p* = 0.0009, Mann-Whitney test).

**FIGURE 6:**
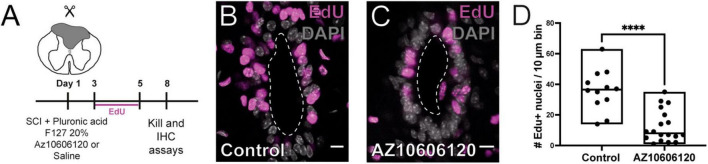
Local blockade of P2X7r reduces injury-induced proliferation. **(A)** Experimental design. **(B)** Numerous nuclei are labeled for EdU in the ependyma of control animals after 8 DPI. **(C)** The number of EdU + nuclei is lower when the hydrogel injected in the lesion was loaded with the selective P2X7r antagonist AZ10606120 (10 μM). **(D)** Boxplot showing the number of EdU + nuclei in control animals was significantly higher than that of mice in which the P2X7r was blocked by AZ10606120 (Mann-Whitney test, *p* < 0.0001) The line of the boxplots represents the median of the values for each sample. ****p < 0.0001. Scale bars: **(B,C)**, 10 μm.

To confirm the role of P2X7r in the reactivation of ependymal cell proliferation induced by injury, we blocked P2X7r *in vivo* by injecting 1 μl (1 μM) of the selective P2X7r antagonist AZ10606120 close to the CC and then performed a dorsal hemisection at the site of the injection. The site of injury was then filled with pluronic acid loaded with 10 μM of AZ10606120 to maintain the blockade of P2X7r by the sustained release of the antagonist (Figure 6A). We found that blockade of P2X7r significantly reduced the proliferative reaction of ependymal cells (Figures 6B–D; *p* < 0.0001, Mann-Whitney test).

## Discussion

The ependyma is a niche of latent progenitors that is activated by injury to contribute new cells to limit the damage (Stenudd et al., 2015; Marichal et al., 2017; Becker et al., 2018). However, the mechanisms that trigger the resumption of the proliferation of ependymal cells and the migration of their progeny are unknown. ATP is one of various “alarmins” released immediately after tissue injury (Gadani et al., 2015) and has been involved as an important signaling molecule after SCI (Cotrina and Nedergaard, 2009; Cheng et al., 2023). We show here that the activation of P2X7r plays a major part in the reactivation of the ependymal stem cell niche mimicking some of the phenomenology elicited by SCI in ependymal cells. Our findings add to the complexity of purinergic signaling in the overall response to SCI and suggest that therapeutic interventions that interfere with P2X7r should take into account the effects on dormant progenitors within the ependyma.

### Purinergic signaling and neural progenitor cells

Purinergic signaling has an important role during development by regulating processes such as progenitor cell proliferation, migration, differentiation and synapse formation (Zimmermann, 2011). For example, ATP released from the retinal pigment epithelium via Cx43 hemichannels induces Ca^2+^ transients in retinal progenitor cells to increase DNA synthesis and proliferation (Pearson et al., 2005). Similarly, during cortical development, the ATP released through Cx hemichannels activates metabotropic P2Y1 receptors in radial glia (RG) generating Ca^2+^ waves by IP3 mediated Ca^2+^ release that propagates among neighboring RG (Weissman et al., 2004). These ATP-induced Ca^2+^ waves regulate RG proliferation, synchronizing the cell cycle of a cohort of progenitors (Weissman et al., 2004). P2Y1 receptors also regulate proliferation of progenitors in the adult subventricular zone (Suyama et al., 2012). In addition to metabotropic P2Yr, P2X7r are also expressed in neural adult progenitor cells (Messemer et al., 2013) and promote cell division both in embryonic stem cells (Glaser et al., 2014) and post-natal hippocampal progenitors (Zou et al., 2012). Ependymal cells lining the ventricles and the CC are normally quiescent but upon brain stroke (Carlén et al., 2009) or spinal cord injury (Mothe and Tator, 2005; Meletis et al., 2008) reenter the cell cycle and generate progeny with the potential to differentiate into different cell types. Functional P2X7r are present in ependymal cells of the brain (Genzen et al., 2009) and SC (Gómez-Villafuertes et al., 2015; Marichal et al., 2016). In ependymal cells of the SC, the selective P2X7r agonist BzATP elicits a slow inward current and an intracellular Ca^2+^ wave that propagates through the cell by a regenerative mechanism implying Ca^2+^ release from internal stores via ryanodine receptors (Marichal et al., 2016). ATP-induced intracellular Ca^2+^ rise has been proposed to couple to Cyclin D to recruit quiescent RG into the cell cycle (Barrack et al., 2014). Thus, it is possible that the resumption of proliferation of ependymal cells by *in vivo* activation of P2X7r reported here is mediated by a general mechanism involving intracellular Ca^2+^ release with modulation of cyclin activity. Although BzATP is a potent agonist of P2X7r, it has also been reported to activate to some degree P2X1, P2X3, P2X5, and P2Y receptors (Pasqualetto et al., 2018). The fact that intraspinal injection of BzATP in P2X7^–/–^ mice did not rescue ependymal cells from quiescence supports the finding that P2X7r are the main purinergic regulator of the cell cycle in progenitor-like cells in the ependyma. This is in line with our previous work showing that while most ependymal cells reacted to BzATP generating Ca^2+^ waves only a small minority (∼8%) responded to the P2Y1 agonist methylthioadenosine disphophate (Marichal et al., 2016). Our findings suggest that unlike bonafide progenitor cells in the developing and adult brain that seem regulated mostly via metabotropic P2Y receptors, cell cycle progression in ependymal cells is unlocked by P2X7r which are activated under pathological conditions due to their low affinity for ATP (Bianchi et al., 1999; Surprenant and North, 2009). Wang et al. (2004) made intraspinal injections of ATP, BzATP and other P2Xr agonists in the dorsal horn and found that whereas ATP and BzATP induced cell death with equivalent potency, agonists for P2X1-5 and P2Y1 did not induce a significant injury, suggesting that in the context of tissue damage P2X7r have a leading role.

Cx are multifaceted regulators that modulate the mitotic activity of progenitors in the developing brain (Elias and Kriegstein, 2008). In the active ependymal stem cell niche, cells are extensively coupled via gap junctions (Russo et al., 2008; Marichal et al., 2012) but uncouple as ependymal cells become dormant during post-natal development (Fabbiani et al., 2020). Upon injury, adult ependymal cells re-coupled with up-regulation of Cx26, an event needed for reactivation of proliferation (Fabbiani et al., 2020). Interestingly, in parallel with the reactivation of proliferation, P2X7r activation promoted the expression of Cx26, suggesting that the P2X7r is upstream of Cx signaling for the regulation of the cell cycle in ependymal cells. It will be interesting to explore whether in the absence of Cx26, the P2X7r retain its ability to restart the proliferation of ependymal cells.

### P2X7r and SCI

ATP signaling plays a key role in different pathologies, with beneficial or detrimental actions (Khakh and Alan North, 2006). It has been proposed that ATP may act as a diffusible “danger signal” to alert about damage and start repair (Dale, 2008). Because their rather low sensitivity (Khakh and Alan North, 2006), P2X7r activation is a distinct feature of the injured CNS (Cotrina and Nedergaard, 2009; Lecca et al., 2016; Territo and Zarrinmayeh, 2021; Yin et al., 2023). Using bioluminescence, Wang et al. (2004) showed that ATP levels increase after SCI in a region spanning about 2 mm of tissue around the lesion epicenter. The increased ATP levels lasted up to 6 h after injury and although the actual concentration of ATP was not reported, neuronal death was reduced by oxATP indicating that ATP release induced by SCI is sufficient to activate P2X7r. Hatashita et al. (2023) used a genetically encoded ATP sensor to measure the ATP concentration around cortical astrocytes and found that spontaneous ATP release from astrocytes *in vivo* reaches the low micromolar range. Interestingly, ATP levels in the cerebrospinal fluid reach micromolar concentrations for several days after SCI (Zhai et al., 2021). Although ATP sources after traumatic injury of the SC remain to be studied in detail, it is likely that the increase in extracellular ATP originates from mechanically induced astrocytic release (Xiong et al., 2018), dying cells and impaired degrading enzymes (Davalos et al., 2005; Gadani et al., 2015). Cytoplasmic ATP concentration is in millimolar range and thus the ATP around dying cells close to the injury site is expected to reach the high micromolar range (Gordon, 1986) needed to activate P2X7r.

P2X7r have been reported ubiquitously in various regions of the CNS in different cell types including neurons, oligodendrocytes, astrocytes, microglia and ependymal cells (Kukley et al., 2001; Yu et al., 2008; Genzen et al., 2009; Gómez-Villafuertes et al., 2015; Marichal et al., 2016). ATP released by trauma activates the inflammasome with the generation of cytokines and chemokines mostly produced by microglia (Gadani et al., 2015) and also astrocytes (Minkiewicz et al., 2013). However, the role of purinergic signaling on ependymal cells within the context of tissue injury remains unknown. We report here that *in vivo* P2X7r activation lead to the resumption of mitotic activity in dormant ependymal cells of the adult SC of mice in a way that closely resembles that induced by injury (Mothe and Tator, 2005; Meletis et al., 2008). P2X7r appear to modulate the cell cycle in different cell types. For example, P2X7r promotes the proliferation of glial progenitor cells in organotypic hippocampal cultures, an effect potentiated by the pro-inflammatory cytokine TNFα (Zou et al., 2012). Microglia in primary hippocampal cultures also proliferate under P2X7r activation (Monif et al., 2009). Similarly, in a mice model of glaucoma, ATP released from activated Müller cells through connexin43 hemichannels acts on P2X7r to induce microglial proliferation (Xu et al., 2022). However, P2X7r have been reported to reduce proliferation of cultured embryonic (Tsao et al., 2013) and adult (Leeson et al., 2018) neural progenitor cells. The fact that ATP reduced the proliferation of adult progenitor cells in P2X7^–/–^ mice suggests that other purinergic receptors may be involved in the regulation of cell cycle and partly explain the conflicting results (Leeson et al., 2018). It is likely that the ultimate effect of P2X7r activation depends on the environmental and intrinsic background of the cell given by the extensive extracellular and intracellular signaling affected by P2X7r (Duan and Neary, 2006). Our results show that P2X7r activation in the absence of injury is sufficient to unlock proliferation of ependymal cells.

Besides the cell cycle, signaling via P2X7r also modulates the differentiation of progenitor cells. In undifferentiated embryonic neural progenitors induced to differentiate into neurons with retinoic acid, P2X7r maintained their proliferation preventing neuronal differentiation (Glaser et al., 2014). However, in neural progenitors cultured from the E15.5 rat brain, P2X7r activation leads to the inhibition of proliferation and the expression of neuronal markers (Tsao et al., 2013), suggesting a complex regulation of cell fate by P2X7r. Indeed, in P19 carcinoma cells P2X7r activation promotes gliogenesis with the expression of the astrocytic marker GFAP (Yuahasi et al., 2012). In the adult SC, GFAP is expressed in a small subset of ependymal cells in the dorsal pole of the CC (Sabourin et al., 2009; Trujillo-Cenóz et al., 2021), but after injury GFAP is expressed in a number of ependymal cells in different quadrants of the ependyma (Meletis et al., 2008; Ren et al., 2017; Trujillo-Cenóz et al., 2021). The fact that BzATP replicated the effects of injury by increasing the number of GFAP + ependymal cells indicates that P2X7r activation is a central mechanism in the phenotypic changes induced by tissue damage in the ependymal stem cell niche. Although the meaning of ependymal GFAP expression induced either by injury or selective P2X7r activation is not clear, it is possible that it reflects a more undifferentiated ependymal cell state that re-entered the cell cycle, similar to neural progenitors in other stem cell niches of the adult (Kriegstein and Alvarez-Buylla, 2009). Because ependymal cells are heterogeneous (Hamilton et al., 2009; Marichal et al., 2012) and seem to contribute differentially to the response of injury (Rodrigo Albors et al., 2023), it will be interesting to explore whether different types of cells react differentially to P2X7r activation.

The ependyma makes an important contribution to limit the extension of a traumatic lesion because ependyma-derived cells migrate toward the lesion site to integrate the glial scar (Meletis et al., 2008; Sabelström et al., 2013). Signaling via ATP regulates migration of intermediate progenitor cells in the subventricular zone (Liu et al., 2008) and oligodendrocyte progenitors (Agresti et al., 2005). ATP released after traumatic brain injury leads to a rapid mobilization of microglia that build a barrier between healthy and damaged tissue (Davalos et al., 2005). Despite P2X7r activation in ependymal cells evoked many injury-induced events, it did not produce a substantial migration of ependyma-derived progeny and is likely to have little functional impact. The lack of effect on migration may be related to the fact that the ATP induced migration of progenitor cells and microglia rely on metabotropic P2Y1r (Davalos et al., 2005; Liu et al., 2008) that are not the main functional purinergic receptor in ependymal cells (Marichal et al., 2016) and are down-regulated after SCI (Gómez-Villafuertes et al., 2015). In line with this interpretation, the migration of neural progenitors from neurospheres is not modulated by P2X7r but facilitated by P2Y1r (Scemes et al., 2003). In addition, it is possible that migration of ependyma-derived cells is driven by chemoattractants other than ATP such as the inflammatory chemoattractant stromal cell-derived factor 1-alpha (Imitola et al., 2004). Future studies should address how the interaction of P2X7r and inflammatory signals impact the biology of progenitors in the ependyma in the context of SCI.

The overall effects of ATP in the context of SCI are complex because of the many cellular targets involved. ATP signaling has been implied in the secondary expansion of tissue damage after SCI (Wang et al., 2004; Peng et al., 2009). For example, P2X7r activate several caspases leading to apoptotic cell death (Ferrari et al., 1999) and P2X7r activation kills motoneurons through a peroxynitrite/FAS-dependent pathway, an effect antagonized by ATP degradation to adenosine (Gandelman et al., 2013). Blockade of P2X7r has been reported to improve functional recovery after SCI by reducing the size of the lesion, microglial activation and reactive astrocytosis (Wang et al., 2004; Davalos et al., 2005). These studies suggest P2X7r may be a relevant target to treat SCI. However, the protective effects of P2X7 blockade remains controversial as another study failed to demonstrate a significant improvement (Marcillo et al., 2012). Our study provides a possible explanation for these conflicting findings because while the blockade of P2X7r would tackle deleterious effects (e.g., pro-inflammatory effects mediated by astrocytes and microglia, neuronal death) it will also block the reaction of ependymal cells to injury and thus the generation of a competent glial scar (Sabelström et al., 2013). The fact that injury still induced some proliferative reaction of ependymal cells in P2X7^–/–^ mice indicates that P2X7r are not the only regulators of ependymal cells. Indeed, acetylcholine via α7*nAChRs has been shown to induce the proliferation of ependymal cells in mice (Corns et al., 2015). In addition, it is likely that receptors other than P2X7r also play a part in regulating the biology of latent progenitors in the ependyma. For example, ependymal cells express the P2Y-like receptor GPR17 which has been proposed as a sensor of tissue damage (Ceruti et al., 2009). More research is needed to understand the intricate mechanisms of purinergic signaling that may affect the response of endogenous spinal progenitors to determine the outcome after SCI.

## Data availability statement

The raw data supporting the conclusions of this article will be made available by the authors, without undue reservation.

## Ethics statement

The animal study was approved by the Comisión de Ética en el Uso de Animales. Instituto de Investigaciones Biológicas Clemente Estable. Avenida Italia 3318, CP11600, Montevideo, Uruguay. The study was conducted in accordance with the local legislation and institutional requirements.

## Author contributions

MF: Conceptualization, Formal Analysis, Investigation, Writing – review and editing. GF: Conceptualization, Formal Analysis, Funding acquisition, Investigation, Project administration, Writing – review and editing. CM: Formal Analysis, Investigation, Writing – review and editing. SV: Investigation, Writing – review and editing. NV: Resources, Writing – review and editing. RER: Conceptualization, Funding acquisition, Project administration, Supervision, Writing – original draft, Writing – review and editing.

## Supplementary material

Supplementary Figure 1 Lack of expression of P2X7r in P2X7r^–/–^ mice. **(A)** Animals were genotyped according to Jacksons Laboratories protocols (primers: TCA CCA CCT CCA AGC TCT TC; TAT ACT GCC CCT CGG TCT TG; GCC AGA GGC CAC TTG TGT AG). Homozygosity was confirmed by electrophoresis (GeneRuler 1 kb Plus DNA Ladder, ThermoFisher Scientific #SM1331; 6X TriTrack DNA Loading Dye, ThermoFisher Scientific #R1161). P2X7r^–/–^ mice (lanes 1–3) showed a single band of 200 bp, while control animals only showed one band of bigger size (approximately 250 bp, lanes 4–5). The results were consistent with data provided by Jackson laboratories (WT = 247 bp, Mutant = 200 bp). Immunohistochemistry for P2X7r in the mouse spinal cord of P2X7r^–/–^ **(B)** and wild-type **(C)** mice.

Supplementary Figure 2 GFAP expression is not affected by BzATP injection in P2X7r^–/–^ mice. **(A–C)**, Immunoreactivity for GFAP in the epicenter of the BzATP injection **(B)** and 2 mm rostral **(A)** and caudal **(C)** from the epicenter. **(D)** Bar graph showing non-significant differences in GFAP expression between the three different regions shown in panel **A** (Kruskal Wallis test, *p* = 0.959, rostral-epicenter; *p* = 0.6725, rostral-caudal; *p* = 0.9999, epicenter-caudal). Scale bars: **A–C**, 10 μm.

Supplementary Figure 3 SCI does not change the GFAP expression in P2X7r^–/–^ mice. **(A, B)** Immunoreactivity for GFAP in the ependyma of injured **(A)** and sham injured **(B)** mice. **(C)** Boxplot showing non-significant differences between the GFAP+ area of both experimental groups (Mann-Whitney test, *p* = 0.3026). The line of the boxplots represents the median of the values for each sample. Scale bars: **A**, **B**, 10 μm.

Supplementary Figure 4 Cx26 expression in the CC after SCI is reduced in P2X7r^–/–^ mice. **(A, B)** Immunoreactivity for Cx26 in WT **(A)** and P2X7r^–/–^ **(B)** mice at 5DPI. **(C)** Boxplot showing a statistically significant lower number of Cx26 puncta in the CC of P2X7r^–/–^ mice compared to that of WT animals (Mann-Whitney test, *p* = 0.0009). The line of the boxplots represents the median of the values for each sample. ****p* < 0.001. Scale bars: **A**, **B**, 10 μm.
