# Supplementary figures and images for: P2X7 receptor activation awakes a dormant stem cell niche in the adult spinal cord
Source: Front Cell Neurosci. 2023 Dec 18;17:1288676. doi: 10.3389/fncel.2023.1288676 (PMC10757934; doi:10.3389/fncel.2023.1288676)

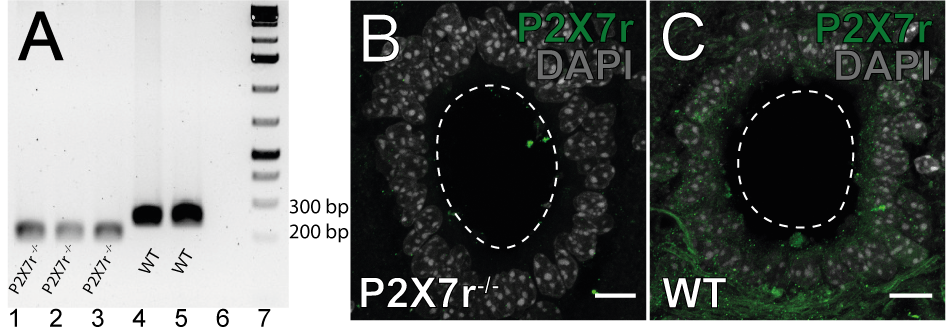

Supplement: Supplementary Figure 1 — Lack of expression of P2X7r in P2X7r–/– mice. (A) Animals were genotyped according to Jacksons Laboratories protocols (primers: TCA CCA CCT CCA AGC TCT TC; TAT ACT GCC CCT CGG TCT TG; GCC AGA GGC CAC TTG TGT AG). Homozygosity was confirmed by electrophoresis (GeneRuler 1 kb Plus DNA Ladder, ThermoFisher Scientific #SM1331; 6X TriTrack DNA Loading Dye, ThermoFisher Scientific #R1161). P2X7r–/– mice (lanes 1–3) showed a single band of 200 bp, while control animals only showed one band of bigger size (approximately 250 bp, lanes 4–5). The results were consistent with data provided by Jackson laboratories (WT = 247 bp, Mutant = 200 bp). Immunohistochemistry for P2X7r in the mouse spinal cord of P2X7r–/– (B) and wild-type (C) mice. [file Image_1.tif]

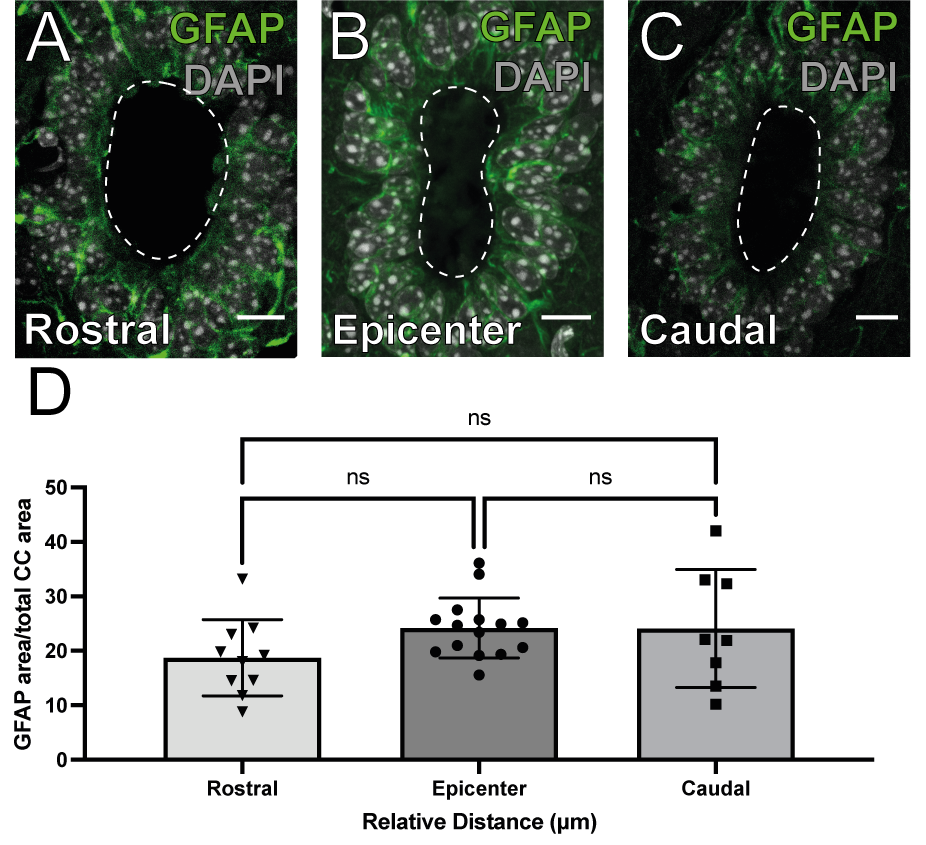

Supplement: Supplementary Figure 2 — GFAP expression is not affected by BzATP injection in P2X7r–/– mice. (A–C), Immunoreactivity for GFAP in the epicenter of the BzATP injection (B) and 2 mm rostral (A) and caudal (C) from the epicenter. (D) Bar graph showing non-significant differences in GFAP expression between the three different regions shown in panel A (Kruskal Wallis test, p = 0.959, rostral-epicenter; p = 0.6725, rostral-caudal; p = 0.9999, epicenter-caudal). Scale bars: A–C, 10 μm. [file Image_2.tif]

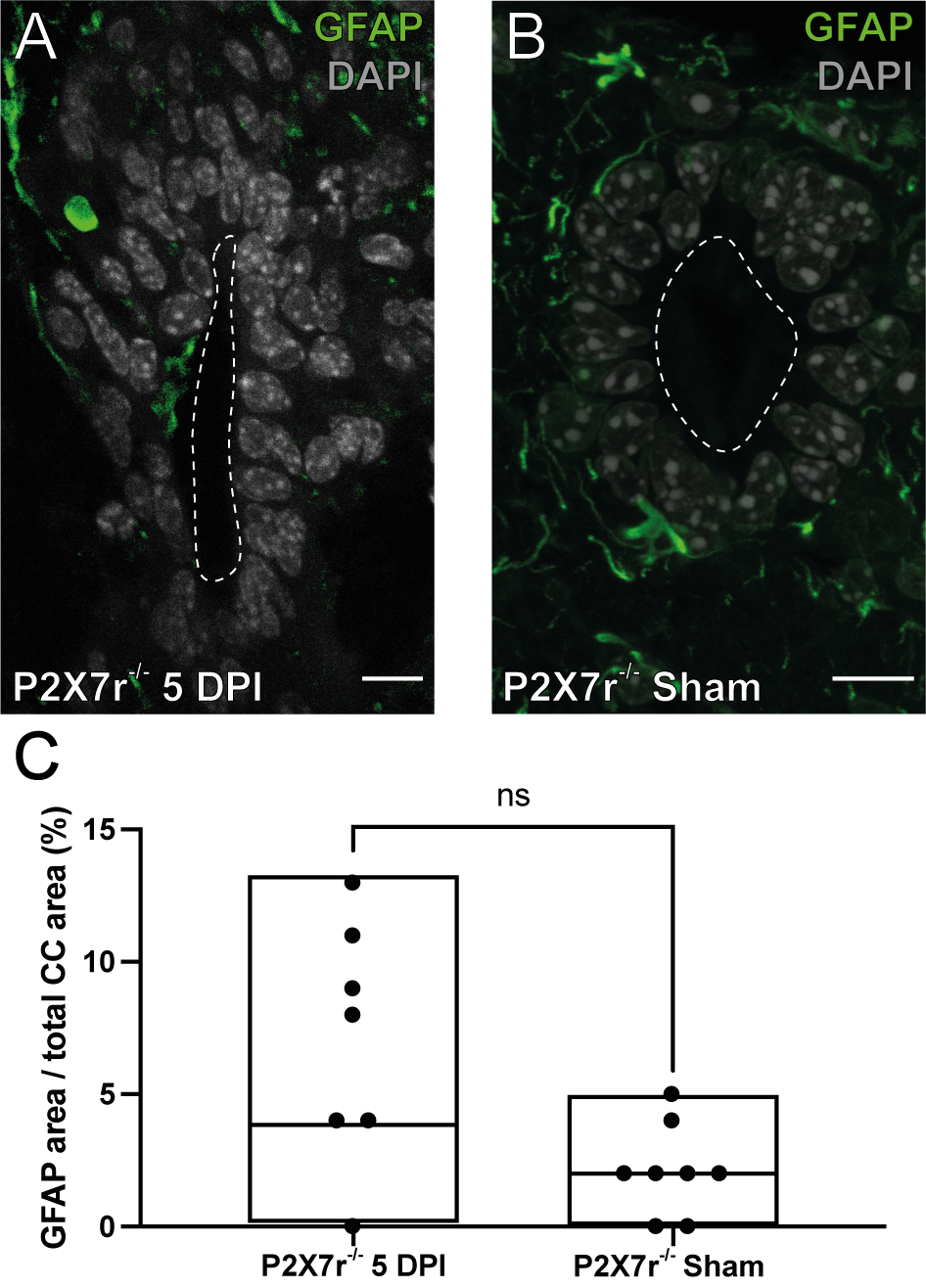

Supplement: Supplementary Figure 3 — SCI does not change the GFAP expression in P2X7r–/– mice. (A, B) Immunoreactivity for GFAP in the ependyma of injured (A) and sham injured (B) mice. (C) Boxplot showing non-significant differences between the GFAP+ area of both experimental groups (Mann-Whitney test, p = 0.3026). The line of the boxplots represents the median of the values for each sample. Scale bars: A, B, 10 μm. [file Image_3.tif]

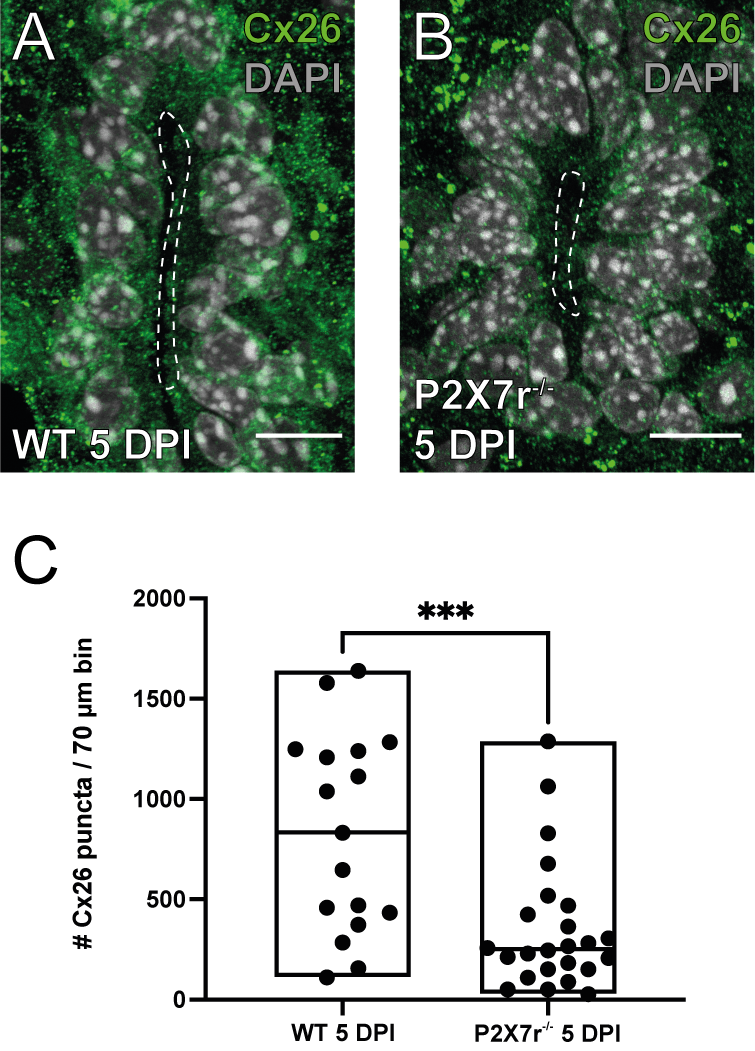

Supplement: Supplementary Figure 4 — Cx26 expression in the CC after SCI is reduced in P2X7r–/– mice. (A, B) Immunoreactivity for Cx26 in WT (A) and P2X7r–/– (B) mice at 5DPI. (C) Boxplot showing a statistically significant lower number of Cx26 puncta in the CC of P2X7r–/– mice compared to that of WT animals (Mann-Whitney test, p = 0.0009). The line of the boxplots represents the median of the values for each sample. ***p < 0.001. Scale bars: A, B, 10 μm. [file Image_4.tif]
